# Fumarate Hydratase-Deficient Renal Cell Carcinoma in a Solitary Kidney: Diagnostic Pitfalls and Onco-Nephrology Constraints in an Older Adult With Chronic Kidney Disease

**DOI:** 10.7759/cureus.116800

**Published:** 2026-09-23

**Authors:** Soumith Sanka, Sai Sushrutha Mudupula Vemula, Ankit Kulkarni, Ujwala Koduru, Borys Hrinczenko

**Affiliations:** 1 Medical School, Michigan State University College of Osteopathic Medicine, East Lansing, USA; 2 Department of Internal Medicine, University of Michigan Health-Sparrow Hospital/Michigan State University (MSU), Lansing, USA; 3 Department of Hematology and Oncology, McLaren Greater Lansing, Lansing, USA

**Keywords:** cabozantinib, chronic kidney disease, fh-deficient renal cell carcinoma, fumarate hydratase, nivolumab, onco-nephrology, solitary kidney

## Abstract

Fumarate hydratase-deficient renal cell carcinoma (FH-dRCC) is a rare, aggressive subtype that is recognized as its own distinct entity in the 2022 WHO classification. It is frequently misclassified as papillary renal cell carcinoma (RCC). Older-onset disease and management in patients with a solitary kidney and chronic kidney disease (CKD) are not well characterized.

A 76-year-old man with a remote right nephrectomy for clear cell RCC and stage 3b CKD presented with two to three years of progressive fatigue. Imaging of the solitary left kidney showed a 4.5-5.2 cm upper-pole mass with bulky retroperitoneal, mediastinal, and hilar adenopathy with multiple pulmonary nodules. Core biopsy findings were initially read as papillary RCC; however, the extensive metastatic burden appeared disproportionate to the relatively modest renal primary.

Re-review demonstrated loss of FH on immunohistochemistry, and tumor sequencing confirmed a pathogenic FH p.G397R missense variant with programmed death-ligand 1 (PD-L1) tumor proportion score 20%, revising the diagnosis to FH-dRCC (cT1bN1M1). Cytoreductive nephrectomy was not pursued because of the patient's solitary-kidney status, baseline CKD, and disease burden. First-line nivolumab plus cabozantinib was selected; therapy was briefly held for immune-related uveitis, then resumed.

This case highlights two important issues: metastatic burden disproportionate to primary tumor size - especially in tumors initially labeled papillary RCC - warrants pathology re-review with FH immunohistochemistry; and a solitary kidney with CKD reshapes the surgical and systemic-therapy calculus in FH-dRCC. Onco-nephrology input is valuable when balancing nephrotoxicity, contrast exposure, and disease-directed therapy.

## Introduction

Fumarate hydratase-deficient renal cell carcinoma (FH-dRCC) is a rare and aggressive renal malignancy caused by pathogenic alterations in the fumarate hydratase (FH) gene. Prior to the 2022 World Health Organization (WHO) 5th edition classification, these tumors were historically grouped under hereditary leiomyomatosis and renal cell cancer (HLRCC) syndrome [1]. Its recognition as a distinct entity follows evidence that a significant proportion of cases carry somatic rather than germline FH mutations, with one series reporting somatic alterations in approximately one-third of patients [2]. The tumor predominantly affects young to middle-aged adults, with a reported median age at diagnosis of approximately 44 years and a moderate male predominance [3].

Most cases arise in the setting of HLRCC, an autosomal dominant hereditary cancer syndrome in which cutaneous leiomyomas represent the most common manifestation, typically appearing in the third decade of life [4]. Clinically, patients often present with advanced disease, manifesting with flank pain, hematuria, or abdominal pain. FH-dRCC exhibits early and aggressive metastatic potential, with nodal and distant dissemination reported at primary tumor sizes as small as 2 to 3 cm [5]. FH-dRCC has a broad histopathological spectrum [6], which may closely mimic other RCC subtypes and make accurate diagnosis challenging.

Late-onset presentations of FH-dRCC are uncommon and may reflect delayed clinical recognition, biologic heterogeneity, or prior tumor misclassification. Advanced patient age can lower diagnostic suspicion, further increasing the risk of a misdiagnosis and delayed treatment. We describe the case of a 76-year-old male who was initially diagnosed with papillary RCC and subsequently found to have FH-dRCC on pathological re-evaluation prompted by an unusually aggressive clinical course. Because morphology alone may not reliably distinguish FH-dRCC from other renal neoplasms, FH immunohistochemistry and, when indicated, molecular profiling are important diagnostic adjuncts when the histologic appearance or clinical behavior raises suspicion for FH deficiency. Hence, accurate diagnosis requires integrating histopathological findings with clinical behavior, and FH-dRCC should remain in the differential diagnosis regardless of patient age [7].

## Case presentation

A 76-year-old male with a history of right radical nephrectomy for clear cell RCC and stage 3b chronic kidney disease (CKD) presented with progressive fatigue and general malaise spanning two to three years. The 2005 right nephrectomy specimen was no longer available for contemporary pathologic re-review, FH immunohistochemistry, or molecular testing. Given his renal history, surveillance imaging was performed. A renal ultrasound demonstrated an abnormal left kidney with an irregularly appearing upper pole lesion measuring 5.2 × 4.6 × 3.3 cm. Concurrent laboratory evaluation revealed a creatinine of 2.0 mg/dL and an estimated glomerular filtration rate (eGFR) of 34 mL/min/1.73 m², consistent with his known stage 3b CKD.

Abdominal computed tomography (CT) (Figure 1) confirmed a large lobulated 4.5 cm left renal mass with extensive retroperitoneal, para-aortic, mediastinal, and hilar lymphadenopathy, as well as pulmonary nodules suspicious for metastatic disease. Subsequent chest CT demonstrated multiple right lung nodules measuring 8 mm in the upper lobe, 15 × 11 mm in the middle lobe, 12 × 9 mm in the perifissural space, and 10 × 7 mm in the lower lobe. Hilar adenopathy was confirmed, right greater than left, with lymph nodes measuring 27 × 22 mm, 29 × 25 mm, and 83 × 33 mm in the hilar, inferior peri-aortic, and left superior retroperitoneal regions, respectively.

**Figure 1:**
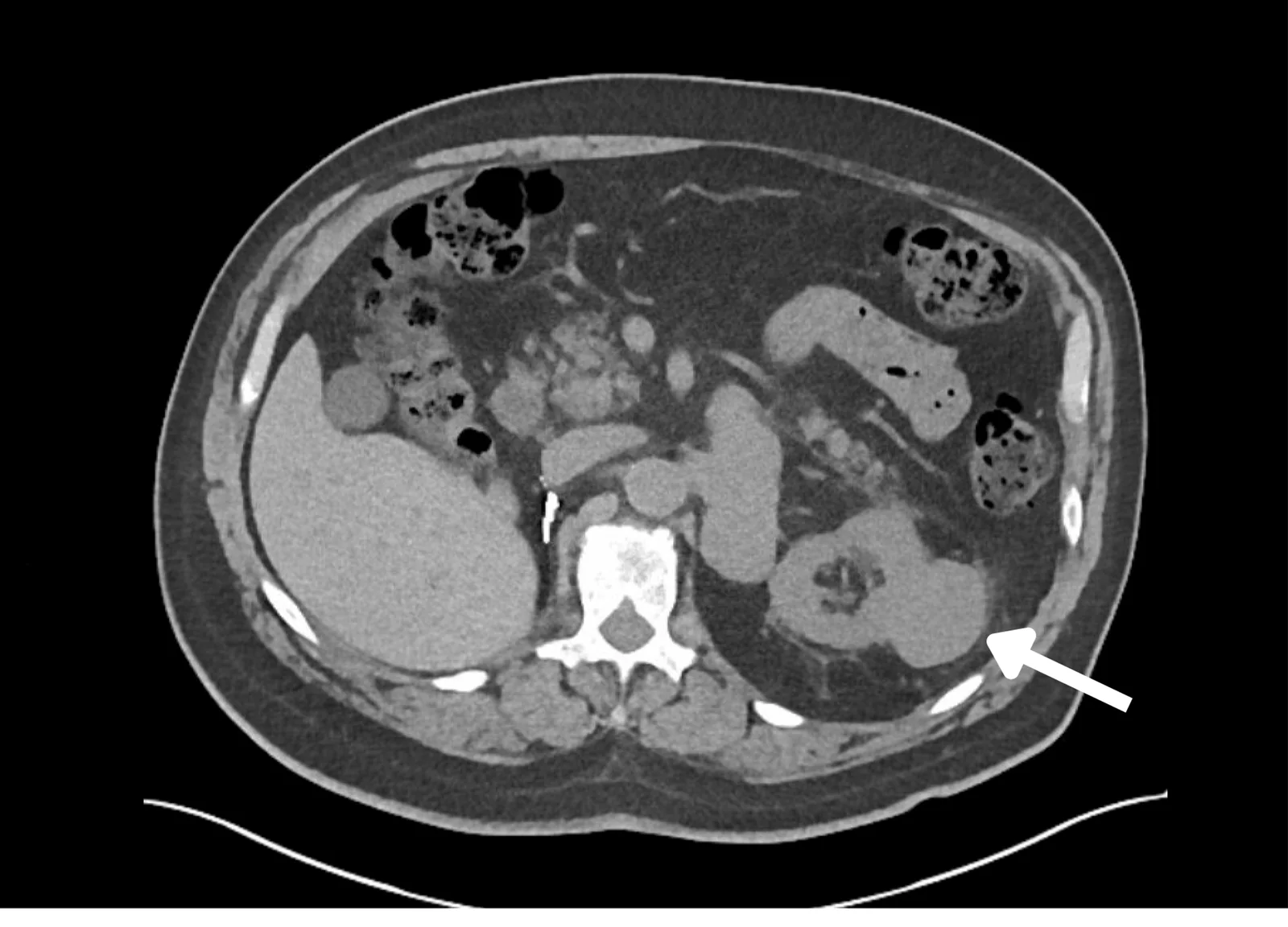
Axial noncontrast CT of the abdomen demonstrating a lobulated upper-pole mass in the solitary left kidney (arrow), measuring approximately 4.5 cm.

Biopsy of the left renal mass was initially interpreted as papillary RCC. Immunohistochemical evaluation demonstrated alpha-methylacyl-CoA racemase (AMACR) positivity, patchy weak carbonic anhydrase IX (CAIX) expression, and negative staining for CK7, CD117, and CEA. Pulmonary evaluation, including lung parenchymal nodule biopsies, bronchial brushings, and bronchoalveolar lavage (BAL), demonstrated no evidence of malignancy. However, fine-needle aspiration of nonregional mediastinal and hilar lymph nodes, including stations 10R and 4R, confirmed metastatic carcinoma of renal origin. These metastatic tumor cells were positive for pancytokeratin AE1/AE3 and PAX8 and negative for TTF-1, supporting renal rather than pulmonary origin. The presence of metastatic renal carcinoma in nonregional thoracic lymph nodes established distant metastatic disease (M1), despite the absence of pathologic confirmation of malignancy in the sampled pulmonary nodules. Given the marked clinical-pathologic mismatch between the relatively modest size of the renal primary and the extensive nodal metastatic burden, the initial diagnosis of papillary RCC was reconsidered and the pathology was re-reviewed.

Follow-up abdominal CT four months after the initial imaging demonstrated interval growth of the left renal mass to 5.1 × 4.7 cm, with an adjacent satellite lesion measuring 2.8 × 1.6 cm and persistent hilar, para-aortic, and retroperitoneal lymphadenopathy. Repeat lymph node sampling confirmed metastatic carcinoma of renal origin, with tumor cells positive for pancytokeratin AE1/AE3 and PAX8 and negative for TTF-1. The updated pathologic interpretation classified the left renal primary and posterior mediastinal lymph node as FH-dRCC. FH immunohistochemistry demonstrated loss of FH expression, and Caris molecular profiling (Caris Life Sciences, Irving, TX, USA) identified a pathogenic FH exon 8 missense variant (p.G397R) with a programmed death-ligand 1 (PD-L1) tumor proportion score (TPS) of 20%. The M1 designation was based on metastatic renal carcinoma involving nonregional mediastinal and hilar lymph nodes. 2-succinocysteine (2SC) immunohistochemistry could not be performed because the available tissue was insufficient for the locally available assay. Although the absence of 2SC staining represents a limitation, the combination of FH loss by immunohistochemistry and the pathogenic FH alteration on molecular profiling supported the diagnosis of FH-dRCC. Detailed architectural and cytologic descriptors from the original H&E interpretation were not available in the records reviewed for this report.

Given the extent of metastatic disease with rapid disease progression, solitary kidney status, stage 3b CKD, and prior contralateral radical nephrectomy, the patient was deemed ineligible for cytoreductive nephrectomy.

Systemic therapy with nivolumab plus cabozantinib was initiated in October 2025. Treatment was temporarily interrupted due to immune-related uveitis, which was managed with topical steroid drops and steroid pellet placement every three months, after which therapy was successfully resumed. Germline genetic testing was subsequently sent to distinguish hereditary from sporadic FH mutation (Table 1, Figure 2). Restaging assessment in February 2026 demonstrated a partial response according to Response Evaluation Criteria in Solid Tumors version 1.1 (RECIST 1.1) [8]. Renal function remained clinically stable, with the most recent eGFR measuring approximately 52 mL/min/1.73 m² compared with 34 mL/min/1.73 m² at baseline. This increase is reported descriptively and should not be interpreted as a treatment effect. Nivolumab was subsequently discontinued because of immune-related adrenal insufficiency. As of May 2026, cabozantinib monotherapy was continued at 20 mg daily.

**Table 1:** Timeline of key events. 2SC: 2-succinylcysteine; Caris: Caris Life Sciences molecular profiling (Irving, TX, USA); CKD: chronic kidney disease; ECOG: Eastern Cooperative Oncology Group performance status; eGFR: estimated glomerular filtration rate; FH: fumarate hydratase; FH-dRCC: fumarate hydratase-deficient renal cell carcinoma; IHC: immunohistochemistry; IMDC: International Metastatic Renal Cell Carcinoma Database Consortium; PD-L1: programmed death-ligand 1; RCC: renal cell carcinoma; RECIST 1.1: Response Evaluation Criteria in Solid Tumors version 1.1 [8]; TPS: tumor proportion score; TTF-1: thyroid transcription factor-1

Time point	Event	Key data
2005	Right radical nephrectomy for clear cell RCC	Solitary left kidney; subsequent stage 3b CKD
Month 0	Onset of progressive fatigue (2-3-year prodrome)	ECOG: 1; baseline eGFR 34 mL/min/1.73 m²
June 2025	Surveillance ultrasound and CT	Left renal mass 4.5 cm; bulky retroperitoneal, mediastinal, hilar adenopathy; pulmonary nodules
August 2025	Renal core biopsy + lymph node sampling	Read as papillary RCC; nodal AE1/AE3+, PAX8+, TTF-1-
September 2025	Repeat CT	Primary 5.1 × 4.7 cm + 2.8 cm satellite; stable adenopathy
October 2025	Pathology re-review prompted by clinical-pathologic mismatch	FH IHC loss; 2SC not performed
October 2025	Nivolumab + cabozantinib initiated	IMDC intermediate risk
October-November 2025	Immune-related uveitis	Topical + intraocular corticosteroids; therapy briefly held, then resumed
December 2025	Caris tumor molecular profiling	FH p.G397R (pathogenic); PD-L1 TPS 20%
December 2025	Diagnosis revised to stage IV FH-dRCC; germline testing sent	cT1bN1M1; germline result pending at the time of submission
May 2026	Nivolumab discontinued; cabozantinib monotherapy continued	Partial response per RECIST 1.1 (February 2026); eGFR ~52 mL/min/1.73 m² (improved from baseline 34); BP 120/70 mmHg; nivolumab discontinued due to immune-related adrenal insufficiency; cabozantinib continued at 20 mg daily
June 2026	Restaging CT	Disease progression with enlarging mediastinal, hilar, and retroperitoneal metastatic adenopathy; enlarging right upper lobe endobronchial and perifissural pulmonary metastases; and enlarging left renal and Gerota’s fascia metastases.

**Figure 2:**
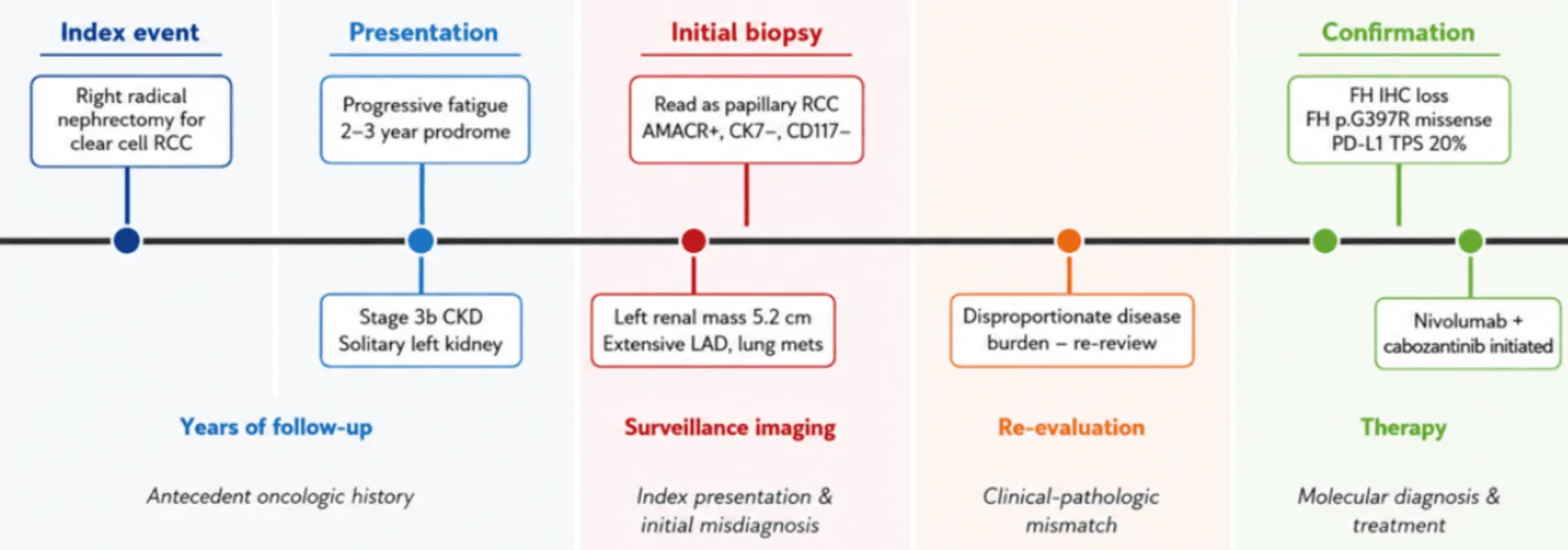
Clinical timeline from the 2005 right radical nephrectomy through detection of the left renal mass, initial diagnosis as papillary RCC, reclassification as FH-deficient RCC following FH immunohistochemistry and molecular profiling, initiation of systemic therapy, treatment-related adverse events, and subsequent response. CKD: chronic kidney disease; FH: fumarate hydratase; IHC: immunohistochemistry; LAD: lymphadenopathy; PD-L1: programmed death-ligand 1; RCC: renal cell carcinoma; TPS: tumor proportion score.

## Discussion

FH-dRCC arises from inactivating mutations in the FH gene on chromosome 1q42.3-q43, with missense mutations being the most commonly reported type [9]. In our patient, a pathogenic p.G397R missense mutation was identified, resulting in inactivation of the fumarate hydratase enzyme, which catalyzes the conversion of fumarate to malate in the tricarboxylic acid cycle. Loss of FH function leads to intracellular fumarate accumulation, which competitively inhibits prolyl hydroxylases that regulate hypoxia-inducible factor 1α (HIF-1α), thereby promoting pseudohypoxic signaling, angiogenesis, and cell proliferation [10]. This oncometabolic reprogramming drives the aggressive phenotype characteristic of FH-dRCC.

Early and accurate diagnosis is critical given the tumor’s propensity for metastasis even at small primary tumor sizes [2,5]. This can be difficult because these tumors can resemble multiple RCC subtypes, displaying papillary, solid, cribriform, tubulocystic, tubular, and sarcomatoid patterns. Papillary architecture is among the most commonly reported predominant patterns. However, mixed architecture involving two or more patterns is frequently observed [6,7,11]. This morphological variability renders histopathological classification alone unreliable, making immunohistochemistry essential for diagnostic workup. However, because FH-dRCC can harbor both somatic and germline mutations and show heterogeneous rather than complete loss of FH expression on immunostaining, immunohistochemistry-based screening by itself can reduce diagnostic sensitivity [12]. Molecular profiling, therefore, plays an important confirmatory role, as demonstrated in our patient, where FH deficiency was identified only after pathological re-review. The delayed recognition in this case reflects several atypical clinical features. Our patient presented at age 76, which, to our knowledge, represents one of the older presentations reported in the FH-dRCC literature, a disease that predominantly affects younger adults. Furthermore, a symptom duration of two to three years prior to diagnosis is atypical for a malignancy known for its rapid and aggressive clinical course and may reflect delayed clinical recognition or underlying biologic heterogeneity. Because germline testing was pending at the time of submission, the identified tumor FH alteration cannot be classified as hereditary or sporadic on the basis of tumor sequencing alone.

Clear cell RCC pathologically appears as nests or sheets of clear cells with arborizing thin-walled vessels, and is most commonly driven by a von Hippel-Lindau (VHL) mutation on chromosome 3p [13]. The prior right nephrectomy specimen from 2005 was not available for contemporary pathologic re-review or FH testing. Consequently, a biologic or hereditary relationship between the patient's prior clear-cell RCC and the current FH-deficient renal malignancy cannot be established, representing a limitation of this case. Papillary carcinoma has historically been divided into types I and II. Type I frequently exhibits arm-level chromosomal gains at chromosomes 7 and 17, while type II rarely demonstrates similar recurrent cytogenetic alterations and represents a heterogeneous group of neoplasms, many of which have been reclassified as FH-dRCC based on contemporary molecular criteria [14]. FH-dRCC relies on FH deficiency as a primary diagnostic criterion, and misclassification remains common, contributing to delays in appropriate treatment.

For localized FH-dRCC, complete surgical resection is generally pursued when feasible because of the tumor's aggressive biologic behavior. In metastatic disease, however, the role of cytoreductive nephrectomy must be individualized according to disease burden, resectability, performance status, comorbidities, and the anticipated morbidity of surgery. In this patient, cytoreductive nephrectomy was not pursued because of extensive metastatic disease, a solitary functioning kidney, baseline stage 3b CKD, and the substantial risk of further renal impairment or dialysis dependence following surgery [15]. Systemic therapy options for advanced FH-dRCC remain limited, as no standardized therapeutic regimen has been established. Several agents and combinations have been investigated, including mTOR inhibitors, nivolumab with ipilimumab, bevacizumab with erlotinib, and nivolumab with cabozantinib [16-19]. Additionally, the combination of sintilimab plus axitinib has demonstrated antitumor activity, though it was associated with high-grade treatment-related adverse events [20]. Among these regimens, nivolumab-based combinations and bevacizumab combined with erlotinib have shown favorable outcomes, including disease remission, whereas mTOR inhibitors alone have not demonstrated meaningful efficacy in tumor reduction or mortality benefit [16,17,19]. Given the patient's metastatic disease, limited surgical options, solitary kidney, baseline CKD, and need for systemic disease control, nivolumab plus cabozantinib was selected after multidisciplinary consideration of his disease burden, renal reserve, comorbidities, and available evidence for systemic therapy in advanced FH-dRCC.

Although FH alterations may occur in the setting of HLRCC syndrome, advanced age should not preclude germline evaluation when a pathogenic FH alteration is identified in a renal tumor. Tumor sequencing alone does not establish germline status. Germline testing and appropriate genetic counseling are therefore important to determine whether a hereditary cancer syndrome is present and, if confirmed, to guide counseling and risk assessment for family members [7,21].

## Conclusions

FH-dRCC is a rare and aggressive renal malignancy that may present with widespread metastatic disease even when the primary tumor remains small. While classically associated with younger patients and HLRCC syndrome, late-onset presentations exist. This case of a 76-year-old male with a prior history of RCC and a solitary kidney, initially misclassified as papillary RCC, highlights the diagnostic challenges of FH-dRCC and emphasizes the importance of a multidisciplinary approach, including comprehensive histopathologic re-evaluation, immunohistochemistry for FH loss, and confirmatory molecular profiling, in achieving an accurate diagnosis. When tumor aggressiveness is disproportionate to primary tumor size, FH-dRCC should remain on the differential diagnosis regardless of patient age or the absence of a known hereditary history. Early and accurate diagnosis has important implications for prognosis and treatment selection and should prompt consideration of germline genetic evaluation and counseling when a pathogenic FH alteration is identified.
